# Influence of the Extraction Solution on the Removal of Heavy Metals from Polluted Soils

**DOI:** 10.3390/ma16186189

**Published:** 2023-09-13

**Authors:** Ioana Monica Sur, Andreea Hegyi, Valer Micle, Timea Gabor, Adrian-Victor Lăzărescu

**Affiliations:** 1 Faculty of Materials and Environmental Engineering, Technical University of Cluj-Napoca, 103-105 Muncii Boulevard, 400641 Cluj-Napoca, Romania; ioana.sur@imadd.utcluj.ro (I.M.S.); andreea.hegyi@incerc-cluj.ro (A.H.); valer.micle@imadd.utcluj.ro (V.M.); 2 NIRD URBAN-INCERC Cluj-Napoca Branch, 117 Calea Florești, 400524 Cluj-Napoca, Romania

**Keywords:** heavy metals, extraction solution, polluted soil

## Abstract

Soil pollution with heavy metals is a problem for the whole geosystem. The aim of the research is to identify new solutions for extracting heavy metals from polluted soils so that they can be further exploited. To this end, investigations of the physicochemical characteristics of the soil sample under study were carried out. Following the analyses, the soil was characterised as lute-coarse sand (UG) with a strongly acidic pH (4.67), a hygroscopicity coefficient (CH = 4.8% g/g), and a good supply of nutrients: nitrogen (N_t_): 0.107%; mobile phosphorus (P_AL_): 6 mg kg^−1^ and mobile potassium (K_AL_): 26 mg kg^−1^, but is low in humus (2.12%). The metal content of the soil was determined by atomic absorption spectrometry (AAS), and the analyses showed high concentrations of metals (Pb: 27,660 mg kg^−1^; Cu: 5590 mg kg^−1^; Zn: 2199 mg kg^−1^; Cd: 11.68 mg kg^−1^; Cr: 146 mg kg^−1^). The removal of metals (Pb, Cu, Zn, Cd, and Cr) from polluted soil by different extraction agents (water, humus, malic acid, chitosan, and gluconic acid) was investigated. Metal extraction experiments were carried out in a continuous orbital rotation-oscillation stirrer at a solid/liquid/ (S/L ratio; g:mL) of 1:4, at two concentrations of extraction solution (1% and 3%), and at different stirring times (2, 4, 6, and 8 h). The yield of the extraction process is very low for all proposed extraction solutions. The maximum values of extraction efficiency are: 0.5% (Pb); 3.28% (Zn); and 5.72% (Cu). Higher values were obtained in the case of Cr (11.97%) in the variant of using humus 3% as an extraction solution at a stirring time of 6 h. In the investigated experimental conditions, the best removal efficiencies were obtained in the case of cadmium (26.71%) when using a 3% malic acid solution. In conclusion, it is considered that, from case to case, the type of extraction solution as well as the nature of the metal influence the mechanism of the depollution process, i.e., the capacity of the fine soil granules to free themselves from the pollutant metal that has adhered to them, and further research is considered necessary in the future.

## 1. Introduction

Soil is a natural resource on which mankind depends for survival and is a significant component of the ecological environment [1]. With the development of industry and agriculture, soil pollution with heavy metals has become a worldwide concern [2,3,4,5]. This problem poses a potential threat to both human health and environmental safety [2,6,7]. Heavy metals are considered priority pollutants in the environment due to their non-biodegradability, toxicity, persistence, and bioaccumulation in the food chain [8,9].

More than 10 million contaminated sites have been identified worldwide, of which more than 50% are contaminated with heavy metals and/or metalloids [10]. The highest levels of soil contamination have been reported in developed countries, i.e., in the United States of America (USA), Australia, Germany, Sweden, and China [11]. Heavy metals account for about 38% of all pollutants identified in European soils [12]. In Romania, the presence of heavy metals, especially copper, lead, zinc, and cadmium, generates the most widespread soil pollution, and its adverse effects are strongly marked. Romanian legislation is aligned with European legislation by Order 756/1997, which imposes certain admissible thresholds for soil pollution [13]. According to Order 756/1997, which regulates the assessment of environmental pollution in Romania, potentially significant pollution is “concentrations of pollutants in the environment that exceed the alert thresholds provided for in the regulations on environmental pollution assessment”. These values define the level of pollution at which the competent authorities consider that a site may have an impact on the environment and determine the need for further studies and measures to reduce pollutant concentrations in emissions/discharges.

Significant pollution means “concentrations of pollutants in the environment that exceed the intervention thresholds laid down in the regulations on the assessment of environmental pollution”, alert threshold is “concentrations of pollutants in air, water, soil or in emissions/discharges, which are intended to alert the competent authorities to a potential environmental impact and trigger further monitoring and/or reduction of pollutant concentrations in emissions/discharges”, and the intervention threshold is “concentrations of pollutants in air, water, soil or in emissions/discharges at which the competent authorities will order risk assessment studies and reduction of pollutant concentrations in emissions/discharges” [14]. In this context, the need to analyse the causes of the degree of pollution as well as the possibilities of remediation is identified. These thresholds represent the pollution thresholds at which the competent authorities warn of the existence, in a given situation, of potential pollution in the air, water, or soil (alert thresholds) or require pollution to be reduced so that pollutant concentrations in emissions/discharges fall to the values laid down in the regulations in force (intervention thresholds). In this context, this type of pollution is identified in Romania in areas such as Baia Mare, Copsa Mica, and Zlatna [15,16]. Research has shown that soils from Copsa Mica are polluted, showing soil metal concentrations of Cu: 69–136 mg kg^−1^, Zn: 962–2191 mg kg^−1^, Pb:1182–1978 mg kg^−1^ and Cd: 30–42 mg kg^−1^ [17], with the soil from Baia Mare having a Cu concentration that exceeded 9.5 times the maximum limits and 4.8 times the alert limits. Pb concentration exceeded 132 times the alert limit and 66 times the action limit, and Zn concentration exceeded 11 and 6 times these limits, respectively [18]. Therefore, this situation is the primary motivation for this research investigation.

Remediation of soils contaminated with heavy metals is essential to mitigate their potential impact on both human health and the ecosystem [2,19]. Various technologies have been developed, tested, and investigated for the extraction of heavy metals from contaminated soils in recent decades, such as solidification/stabilization, soil washing, soil amendment, phytoremediation, and electrokinetic methods [20,21,22,23,24,25,26]. These techniques commonly used to remediate heavy metal-contaminated sites are effective but often require high costs [14], are labourious and complicated [27], have limited applicability to soil remediation [28,29], do not allow natural soil recovery, and are time-consuming or involve extensive labour [30,31]. Among them, soil washing is one of the most effective treatment technologies with a short application time and is relatively cheap for the decontamination of heavy metal-polluted soils [1,25,31,32,33,34,35,36,37,38]. Soil washing involves the extraction of heavy metals by chemical solutions, which is a promising method if the applied extraction agent does not alter the soil matrix and its characteristics [39]. Heavy metals tend to be chemically or physically attached to the finer soil particles, which are in turn bound to the coarser particles, thus trapping heavy metals in the finer soil particles [40]. When washing the soil with different washing agents, the influence mechanism between the metals and the substance that is in the composition of the extraction solution is taken into account. Thus, the type of interaction depends on the type of metal, its chemistry, and the pH of the extraction solution [41]. For example, the amine (NH_2_) and hydroxyl (OH) groups of chitosan are able to adsorb metals through several mechanisms, including chemical interactions (such as chelation) and electrostatic interactions (e.g., ion exchange or ion pair formation) [41,42,43]. The ability of chitosan is pH-dependent [44]. The amino group in the chitosan structure binds metal cations at a near-neutral pH. At low pH values, the amino group in the chitosan structure protonates, becomes positively charged, and is therefore able to bind anions by electrostatic attraction or ion exchange [43,45].

When analysing the structural complexity of humic substances, we find that they can be oxidised by strong oxidising agents, act as reducing agents, take part in protolytic, ion exchange, and complexation reactions, and engage in hydrogen bonding [46]. Thus, humic substances can interact with virtually all chemicals released into the environment, including heavy metals, chlorinated or petroleum hydrocarbons, pesticides, dyes, actinides, etc. [46]. The mechanism of the binding of metal ions by humic substances is complex; there is no clear explanation for this interaction, and there are conflicting opinions among researchers about the mechanism of the interaction between metals and humic acids. The main agents responsible for the binding of metal cations are humic acids and their functional groups [47], and carboxyl groups are responsible for binding metal ions [48]. Other researchers indicate that both carboxyl and phenolic groups are involved in this interaction [49]. Even the possibility of metal ions binding to nitrogen atoms in humic acids has been suggested [50]. On the other hand, the availability of functional groups of humic acids is strongly influenced by pH [47].

Various studies have demonstrated the efficacy of using different washing agents (e.g., hydrochloric acid—HCI, sulfuric acid—H_2_SO_4_, nitric acid—HNO_3_, oxalic acid, citric acid, saponin, ethylenediaminetetraacetic acid—EDTA, nitrilotriacetic acid—NTA, and ethylenediaminedisuccinic acid—EDDS) [51,52,53], but also the adverse effects that may result from their use, such as the release of additional contaminants into the environment [39] and changes in soil pH, soil structure, soil productivity, soil microorganism activity, or soil microbial health [54,55].

The important issue in the development of heavy metal-contaminated soil treatment technology by scrubbing is the identification of a scrubbing reagent with a high pollutant removal rate, low toxicity [1,2], low deterioration of soil properties [56], low cost, and high biodegradability [54,57]. In this regard, some substances have been highlighted through which the extraction of metals from polluted soils can be achieved: water, humic substances, malic acid, D-gluconic acid, and chitosan. *Water*, which is economical and easily accessible, can clean contaminants that are in a soluble state. Heavy metals are poorly soluble, and washing them with water alone does not remove a significant number of heavy metals [58]. *Humic substances* have a higher affinity for Pb^2+^ and Cu^2+^, and the higher the pH of the solution, the higher their adsorption [26,59]. *Malic acid* (MA) can form soluble complexes with heavy metals, facilitating their migration into the liquid phase [2,60], with properties of high biodegradability, low toxicity, low price, and being environmentally friendly [1,61,62,63,64]. Studies have shown that by using malic acid, heavy metals are extracted from polluted soils with an efficiency of 50.8%, according to literature reports [65], or even 63.39–88.65%, as reported by Zhao [1], for the case of Pb. For the case of Cd extraction efficacy, values vary, influenced by the concentration of the malic acid solution used, from 1.91–6.22% according to Ma et al. [5], 39.86% as reported by Li et al. [66], or even 60% according to Han et al. [65]. Chromium concentration reduction occurs under low pH conditions, while in the case of Cu, increasing pH has a favourable effect on soil depollution [67]. Consistent with the above, other researchers have also shown that pH values can affect the mobility of heavy metals in leaching solutions [68,69]. Additionally, research has shown that washing with malic acid did not alter soil microstructure [1], and washing efficiency improved significantly with increasing washing times [66]. Therefore, malic acid solutions are regarded as promising washing agents to remediate contaminated soil [1]. *D-gluconic acid* (D-GA) is a polyhydroxy carboxylic acid that is non-toxic and readily biodegradable, and has great potential for the activation of heavy metals in soil [65,70,71,72]. The ability of D-gluconic acid to leach heavy metals from polluted soil is strongly influenced by the pH value. The leaching capacity is very low at a near-neutral and slightly basic pH. It increases greatly for pH values in the range 12.0–13.0 (Pb: 80%; Cu: 84%; Zn: 70%; Cd: 63%; and Cr: 60%) [72]. Results indicated that the use of D-gluconic acid (D-GA)-based solutions showed extraction rates of 34.4% for Cd and 30.0% for Pb [65]. *Chitosan* is a common and biodegradable chelating polymer [73,74], with the potential to extract metals from polluted soils [75,76]. The efficiency of heavy metal removal by soil washing using chitosan as a washing agent is influenced by the liquid/solid ratio, the concentration of washing solution in chitosan, the duration of soil washing, the pH of the washing solution, the type of pollutants present in the soil, etc. [26,77,78]. Chitosan has been shown to be effective for the removal of zinc (Zn) from soil [78]. The maximum adsorption capacity of cadmium (Cd) was reported at pH 5.0–6.0 [79], with a removal efficiency of 93.76% [80] and 100%, respectively [41].

The removal efficiency of heavy metals from contaminated soils also depends on the nature of the soil and the type of heavy metals [2,57].

The main objective of the present work was to select a suitable solution for the efficient removal of heavy metals from soil by washing. For this purpose, five washing solutions were chosen: water, humus, malic acid, chitosan, and gluconic acid for treating heavy metal-contaminated soil. The influence of soil physicochemical properties and parameters of the washing process (solid/liquid ratio, concentration of extraction solution, stirring time) was experimentally investigated, and the efficiency of the extraction process was determined. The results obtained from this study might be useful for the choice of washing agent and for the remediation of heavy metal-polluted soil by applying soil washing technology. Considering the fact that a number of decontamination techniques are currently used, the aim of this study is to identify potential solutions that are more environmentally friendly and economically sustainable and that also allow multiple repeated treatments, oriented towards the possibility of reintegrating soils into the usual circuit. The approach of these cheaper and more environmentally friendly treatment solutions, aligned with the worldwide trends in action to reduce pollution from all points of view and as close to the source as possible, is all the more appropriate as, in this case, the soil in the investigated area shows a very high contamination with heavy metals [81,82], and this soil needs to be brought to lower concentrations in the first phase. This approach is also motivated by the fact that this soil cannot be used in agriculture due to the high concentrations of metals and that the use of amendments is necessary to bring pH to an optimal value [83].

## 2. Materials and Methods

### 2.1. Materials

In general, soil pollution is the accumulation of toxic chemical compounds, pathogens, radioactive materials, and heavy metals, which leads to negative effects on the conditions for the growth and life of plants, animals, and humans. Based on this definition, a soil sample was taken from a known polluted area in Romania according to the Romanian standard STAS 7184/1-84 [84], which describes how it is collected for a soil sample, which was then processed for physicochemical analysis according to ISO 11464:1998 [85]. The soil sample analysed by the sampling location (a north-western area of Romania, in the immediate vicinity of the copper and lead mining area) was judged to be representative for the category of polluted mining and adjacent soils.

### 2.2. Soil Analysis Methods

Before soil treatment, soil properties were determined. The pH determination was carried out by the potentiometric method according to the methodological rules laid down in STAS 7184/13-88 [86] and SR ISO 10390:1999 [87] using the portable pH meter HANNA pH meter (Hanna Instruments, Woonsocket, RI, USA). Soil pH was determined in soil/water extract 1:10 (*w*/*v*).

The *structural condition* was assessed by the Hénin–Fedoroff method by determining the content of hydrostable macroaggregates with a diameter > 0.25 mm (AH) and the content of hydrostable microaggregates with a diameter less than 0.02 mm (D) resulting from the mechanical separation of aggregates [88]. Based on these determinations, the structural instability index (IS), a synthetic indicator comprising both macrostructural and microstructural data, was calculated [89,90]. The calculation of the index was performed according to Equation (1):

IS = D/(AH − 0.9n_g_), (1)

where IS is the structural instability index; D—content of hydrostable microstructural elements < 0.02 mm in diameter, in %; AH—content of hydrostable macrostructural elements; and n_g_—coarse sand content, in %.

*Texture determination* was performed by separating the grain size fractions. Soil macrotexture was determined by the sieving method for separating particles larger than 2 mm (forming the skeleton) and coarse sand (2–0.2 mm) and by the sedimentation method in a still column of water (Kubiena pipetting method/Kacinski method), based on the theory that the velocity of falling particles in a liquid depends on their size for separating grain size fractions smaller than 0.02 mm [88,91]. Subsequently, the particle size fractions of fine sand (0.2–0.02 mm), coarse dust (0.02–0.01 mm), fine dust (0.01–0.002 mm), and clay with aid (<0.002 mm) were calculated [91,92].

The *hygroscopicity coefficient* (CH) was determined by the direct Mitscherlich method according to STAS 7148/6-78 [93]. This method consists of saturating the soil sample with water vapour in a vacuum over a 10% sulphuric acid solution, which gives a relative humidity of 94.3% at a temperature of 20 °C. The hygroscopicity coefficient was determined using Equation (2) [93]:

CH = (m_1_ − m_2_)/m_2_ × 100, (2)

where CH is the hygroscopicity coefficient, in %; m_1_—mass of the soil sample after removal from the desiccator, in g; and m_2_—mass of the soil sample after drying, in g.

The *organic carbon* was determined by wet oxidation and titrimetric determination according to the Walkey–Black method modified by Gogoasa. The principle of the method consists of oxidising organic carbon in humus with potassium dichromate in the presence of sulphuric acid, then titrating the excess oxidant with a Mohr salt solution in the presence of a redox indicator [94].

*Humus* determination was performed according to STAS 7184/21-82 [94]. Organic carbon, the main constituent of humus, was oxidised in the presence of sulphuric acid at 100 °C for 30 min. The humus content was estimated directly from the organic carbon content by multiplying it by a coefficient (1.7241), based on the finding that the average percentage carbon content of humus is 58% [95]. The carbon/nitrogen ratio (C/N) was considered to assess the quality of humus.

The determination of *micronutrients* (total nitrogen, phosphorus, and potassium) was necessary to perform a comprehensive soil analysis.

*Total nitrogen* (TN) was determined by the Kjeldahl method according to the methodological rules laid down in STAS 7184/2-85 [96] and processed according to the rules of SR ISO 11261/2001 standards [97]. *Soil mineralization* was determined by boiling in concentrated sulfuric acid in the presence of a catalyst, followed by distillation in an alkaline medium, absorption of ammonia released in a boric acid solution, and titration with sulfuric acid. The total nitrogen content together with the amount of organic carbon in soils are the elements for calculating an important pedogenetic index, the C:N ratio [93].

Determination of *mobile phosphorus* (accessible P) was performed by the Egner–Riehm–Domingo (P_AL_) method, calorimetrically, with extraction in ammonium lactate acetate (AL), according to STAS 7184/19-82 [98] at pH = 3.75. The extractable phosphorus content with AL reagent, relative to air-dry soil, was calculated according to Equation (3) [98]:

phosphorus (P) = (C × r)/m,   [mg kg^−1^] (3)

where C is the content of phosphorus in the calorimetric sample determined from the calibration curve, in μg; r—the ratio between the volume of solution used for extraction and the volume of extract pipetted for the determination of phosphorus; and m—the mass of soil analysed, in g.

*Mobile potassium* (accessible, exchangeable K) in soil was determined in acetate-ammonium lactate (AL) extract with the Egner–Riehm–Domingo (K_AL_) method by flame photometry using various extraction methods followed by flame photometric dosing.

Analyses of cation exchange properties were carried out, according to STAS 7184/12-79, as follows [99].

The sum of *basic exchange cations* (SB) was determined by the Kappen method with 0.1 n KCl according to STAS 7184/12-79 [99] to find out the exchangeable cation content of alkaline and alkaline earth elements (K^+^, Na^+^, Ca^2+^, and Mg^2+^) [100,101].

*Total exchange acidity* (TEA) was determined by leaching to exhaustion with a buffered solution at pH = 8.3 of potassium acetate, solution 1 n, according to STAS 7184/12-79 [99].

The *total cation exchange capacity* (T) was determined by calculation, summing the sum of the basic exchange cations (SB) and the total exchange acidity (SH).

The *content of H^++^ ions* was calculated according to Equation (4) [99]:

H = A_s_ − Al^3+^,   [me/100 g] (4)

where A_s_ is extractable exchange acidity and Al^3+^—content of Al^3+^ ions.

### 2.3. Determination of Soil Mineral Phases

X-ray diffraction (XRD) is required to identify the mineral phases in the soil composition. The diffraction lines specific to each mineral are digitally recorded as a diagram. The analysis was carried out with the SHIMADZU XRD-6000 X-ray diffractometer (Shimadzu Company, Tokyo, Japan) with a Cu anticathode. For each diffraction, the range of analysis was 2θ = 4–60. Crystal phases and compounds were identified using Match 1.0 software equipped with the PDF2 database package, Crystal Impact Company, Bonn, Germany.

### 2.4. Determination of Soil Metal Concentration

Soil metal contents were determined by atomic absorption spectrometry (AAS) with a SHIMADZU AA-6800 spectrometer (Shimadzu, Tokyo, Japan) using the aqua regia di-gestion. The entire soil sample was dried, ground to a fine powder, and sieved through a 100 μm sieve [102]. This was undertaken in order to have a homogeneous soil sample so that the entire soil sample is subjected to the mineralization process and because it is known that grinding increases the specific surface area, i.e., an improvement of the soil/extraction solution contact surface occurs. Three grammes of soil were weighed into a beaker, to which 7 mL of HCl and 21 mL of HNO_3_ were added. After 3 h of mineralization, the supernatant was filtered through a 0.45 μm pore size filter into a 100 mL volumetric flask, filled to the mark with distilled water, and then subjected to heavy metal analysis by AAS. Investigations were carried out under constant conditions of temperature, actual air humidity, and ventilation: T = (27 ± 1) °C and RH = (65 ± 2)%, without forced ventilation of ambient air.

In order to determine the degree of soil pollution, the results obtained were compared with the values established in Order 756/1997 [13] concerning normal values, alert threshold values, and intervention threshold values (Table 1).

### 2.5. Preparation of Extraction Solutions

Five extraction solutions were used in the experiment: water (A); chitosan (C) extracted from shrimp shells, supplied by the company Sigma-Aldrich, Saint Louis, MO, USA; humus (H) obtained by alkaline extraction from leonardite of German origin found commercially under the name Powhumus WSG-85, supplied by the company Humintech GmbH (Grevenbroich, Germany); malic acid (AM), supplied by Penta Chemical (Wuchterlova, Czech Republic); gluconic acid (AG), supplied by the company Alfa Aesar GmbH (Karlsruhe, Germany). The substances used (chitosan, humus, malic acid, and gluconic acid) are commercially available in solid form, from which they have been chemically modified.

Each substance was weighed with an electronic balance with a precision of 0.001 g, 10 g (i.e., 30 g, to which 1000 mL of distilled water was added). This resulted in two extraction solutions of 1% and 3% concentration, respectively, to see if a lower concentration is sufficient to make the remediation technology as environmentally friendly and financially feasible as possible. These solutions were used as extraction agents for the remediation of polluted soils.

### 2.6. Metal Extraction

In order to extract metals (Pb, Cu, Zn, Cd, and Cr) from polluted soil, a series of experiments were carried out using the extraction solutions presented in Section 2.5.

Experiments on the possibility of heavy metal extraction were carried out at a laboratory scale under constant conditions of temperature, real air humidity, and ventilation: T = (27 ± 1) °C, RH = (65 ± 2)%, without forced ventilation of the ambient air.

Four sets of samples were prepared in 250 mL Erlenmeyer dishes for each of the extraction solutions used, as follows: 5 g of soil was weighed out, and the extraction solution was added to the soil, keeping the solid:liquid S/L ratio constant (1:4). The coding of the samples is shown in Table 2. The samples thus prepared were placed in a continuous orbital rotation–oscillation stirrer at 200 oscillations/min at a stirring time of 2 h, 4 h, 6 h, or 8 h, with one set of samples for each stirring time duration. Subsequently, the soil was separated from the extraction solution by filtration, and the leachate was analysed by atomic absorption spectrometry to determine the metal concentrations (Pb, Cu, Zn, Cd, and Cr). To ensure repeatability and reproducibility, results were recorded as an average of three successive measurements.

During the extraction period, the pH evolution was also monitored, so the pH was determined for each sample. The pH was determined using a Hanna HI 9829 Multiparameter (Hanna Instruments, Woonsocket, RI, USA).

### 2.7. Extraction Process Efficiency

Based on the recorded experimental results, the extraction process efficiency of heavy metals was determined on a laboratory scale, depending on the parameters studied (dose of soil, wash solution ratio, concentration of wash solution, stirring time).

The removal efficiency from heavy metal-contaminated soil, i.e., this extraction efficiency (ƞ), was determined as the mass percentage between the amount of metal extracted, determined according to Section 2.6, and the amount of metal available in the analysed soil, determined according to Section 2.4.

## 3. Results and Discussions

### 3.1. Soil Characterization

**Figure 1:**
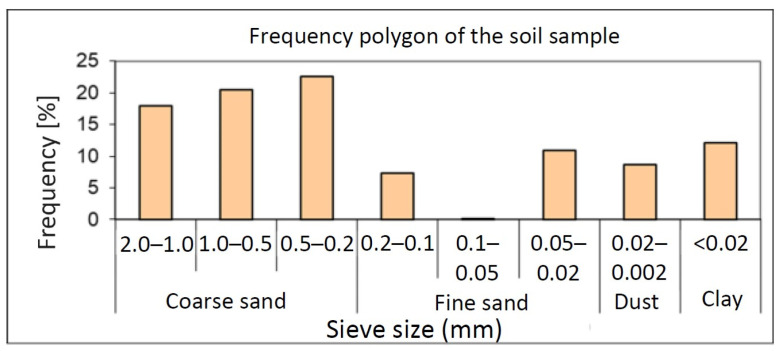
Frequency polygon of the soil sample.

The properties of the soil used prior to treatment for heavy metal extraction are shown in Table 3. Based on the grain size composition and the values obtained for the hygroscopicity coefficient (CH = 4.8% g/g), the soil was characterised as coarse loamy sand (UG) with a strongly acidic pH (4.67). The determinations showed that the sample has a good supply of nutrients. N_t_: 0.107%; P_AL_: 6 mg kg^−1^; K_AL_: 26 mg kg^−1^ but is low in humus (2.12%). Soil textural analysis (Figure 1) indicated that the soil sample shows a sandy-clay macrotexture.

Following the tests carried out on the total alkaline-earth carbonate content (K^+^, Na^+^, Ca^2+^, and Mg^2+^) (Table 4), it was found that in the sample analyzed there are no carbonates with low ferocity since the sum of basic exchange cations (SB) is extremely low (1.63), and the degree of saturation in basic cations (V = 15.2 me) revealed that the sample analyzed is oligomezobasic.

The soil sample shows values that fall below the total cation exchange capacity (T = 10.7 me) at pH = 8.36 to a low class [102,103], having a total soluble salt content of 10.70 mg.

The soil sample taken shows physicochemical characteristics that are similar to those of other samples taken from the same area, for which research has been reported in the literature. The soil is strongly acidic. pH = 4.4 [102]; 3.77–4.6 [104] with a humus content of 0.33% [102]; 0.66–4.44 [104]; Nt = 0.048% [102]; 0.07–0.23 [104]; P = 10 mg kg^−1^ [102]; and C_org_ = 0.38–2.57% [104]. The SB value (1.63 mg), however, is lower than that reported in other studies: 15.38 mg [102] or 29.1–68.2 [104].

### 3.2. Determination of Mineral Phases

**Figure 2:**
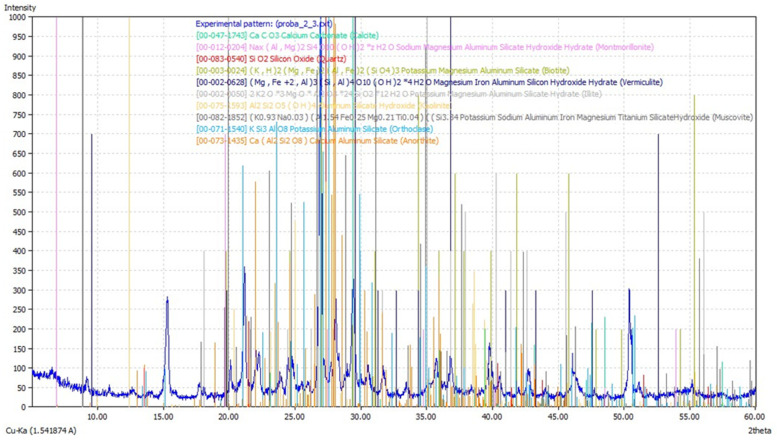
Overall diffractogram of the sample (where * represents ×).

The mineral phases identified in the soil sample by X-ray diffractogram analysis are: clay minerals (kaolinite (Al_4_), montmorillonite (Na,Ca)_0.33_(Al,Mg)_2_(Si_4_O_10_)(OH)_2_·nH_2_O), illite (K,H_3_O)(Al,Mg,Fe)_2_(Si,Al)_4_O_10_[(OH)_2_,(H_2_O)] and vermiculite (Mg,Fe^2+^,Fe^3+^)_3_[(Al,Si)_4_O_10_](OH)_2_·4H_2_O), micas (biotite K(Mg,Fe)_3_(AlSi_3_O_10_)(F,OH)_2_ and muscovite Kal_2_(AlSi_3_O_10_)(F,OH)_2_) feldspars KAlSi_3_O_8_–NaAlSi_3_O_8_–CaAl_2_Si_2_O_8_, quartz SiO_2_, and calcite CaCO_3_ (Figure 2).

The mineral phases of the soil investigated in this work are similar to other studies carried out on soils from the same study area, and the existence of montmorillonite and allophane in the clay fraction associated with hydromuscovite, feldspar, and quartz was revealed by X-ray diffraction determinations [82].

### 3.3. Determination of Soil Metal Concentration

The concentration of heavy metals is presented in Table 5, and the values are compared with the normal value, the alert threshold, and the intervention threshold according to Order No. 756/1997 [13]. All five metals exceed the normal value and the alert threshold for the category of sensitive soils. As regards the intervention threshold, the samples show excesses, with the exception of chromium (146 mg kg^−1^), which has values lower than this intervention threshold of 300 mg kg^−1^. If we analyse the concentration of metals according to the category of less sensitive soils, the same trend is observed for sensitive soils. The concentration of lead is very high (27,660 mg kg^−1^), exceeding the intervention threshold for less sensitive soils (1000 mg kg^−1^) by almost 28 times, while copper (5590 mg kg^−1^) exceeds the intervention threshold for less sensitive soils (500 mg kg^−1^) by 10 times. Zinc and cadmium show lower concentrations but still exceed the intervention threshold for less sensitive soils.

### 3.4. Metal Extraction from Soil

During the extraction process, it was observed that the pH was constant in each extraction solution regardless of the stirring time of the extraction solution. The pH of the extraction solution increases with the concentration of the extraction solution in the case of chitosan and humus, while in the case of malic acid and gluconic acid, it decreases as the concentration of acid used increases (Table 6). In this context, and in agreement with some references in the literature [83], it is appreciated that the pH of the extraction solutions, although it is in an acid/neutral zone, could induce a slight acidification of the treated soil, an acidification that could find potential benefits if exploited in agriculture to favour the development of plant species that prefer more acidic soils.

**Figure 3:**
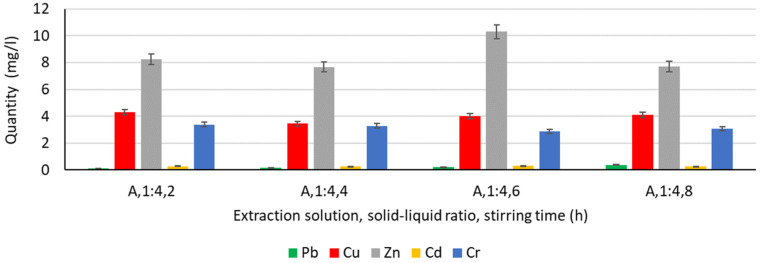
Extraction of Pb, Cu, Zn, Cd, and Cr in water.

**Figure 4:**
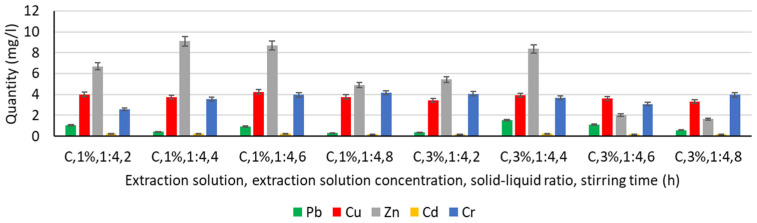
Extraction of Pb, Cu, Zn, Cd, and Cr in a chitosan solution.

**Figure 5:**
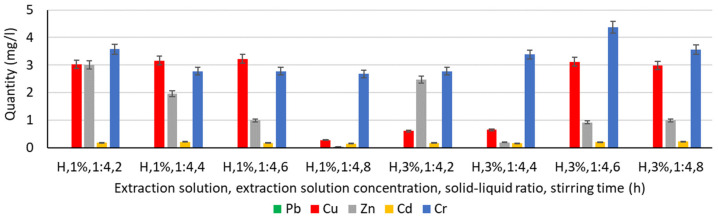
Extraction of Pb, Cu, Zn, Cd, and Cr in a humus solution.

**Figure 6:**
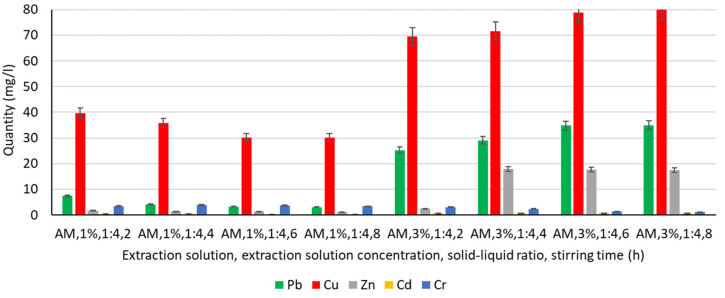
Extraction of Pb, Cu, Zn, Cd, and Cr in a malic acid solution.

**Figure 7:**
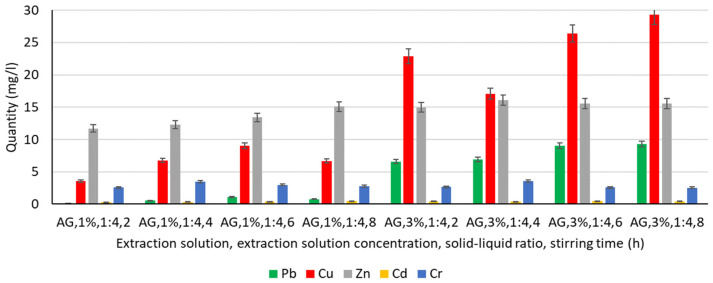
Extraction of Pb, Cu, Zn, Cd, and Cr in a gluconic acid solution.

In order to analyse the influence of the extraction medium, i.e., the type and concentration of the solution used for the extraction of Pb, Cu, Zn, Cd, and Cr, the following solutions were used: water, chitosan solution 1%, chitosan solution 3%, humus solution 1%, humus solution 3%, malic acid solution 1%, malic acid solution 3%, gluconic solution 1%, and gluconic solution 3%. The experimentally obtained results are shown in Figure 3, Figure 4, Figure 5, Figure 6 and Figure 7.

#### 3.4.1. Extraction of Heavy Metals from Water

In the case of using water as the extraction medium (Figure 3), it can be said that the most favorable results are obtained for Zn, followed by Cu and Cr. For Pb and Cd, the results indicate a high probability that the liquid medium, water, is not favourable for extraction.

#### 3.4.2. Extraction of Heavy Metals from Chitosan Solution

In the case of using chitosan solutions of 1% and 3% concentration as extraction media (Figure 4), it can be said again, similarly to the case of using water, that the most favourable results are obtained for Zn, followed by Cu and Cr. For Pb and Cd, the results indicate an increased probability that this liquid medium, chitosan, is not favourable for extraction. However, compared to the situation using water, there is an improvement in the increase in Pb extraction.

For the same concentration of chitosan solution, the experimental results indicate a slight influence of the stirring hardness. Thus, in the case of a 1% chitosan solution, a maximum of Zn extraction is observed for a stirring time of 4 h and a minimum for a stirring time of 8 h. In the case of Cu and Cd, increasing the stirring time does not bring significant variations in terms of the amount of metal extracted, and in the case of Cr, there is a slight improvement in the extraction process with increasing stirring time (from 2.58 mg L^−1^ for 2 h stirring time to 4.17 mg L^−1^ for 8 h stirring time).

In the case of using 3% chitosan solution, the same maximum Zn extraction is observed for a stirring time of 4 h, as well as the same lack of influence of stirring time on Cu and Cd extraction. On the other hand, the trend of improving Pb extraction with increasing stirring time is not maintained.

Analysing the amount of metal extracted for the same element (Pb, Cu, Zn, Cd, or Cr), but under conditions of different concentrations of chitosan solution (1% or 3%), it can be said that:

- In the case of Pb, for stirring times of 4 h, 6 h, and 8 h, increasing the concentration of the chitosan solution induces an increase in the amount extracted even by 200% (stirring time 4 h);
- Cu extraction is slightly reduced by 5–15% with increasing chitosan solution concentration for all stirring times;
- In the case of Zn, increasing the concentration of the chitosan solution reduces the extraction process, regardless of the stirring time. In the case of longer stirring, there is even a significant reduction of Zn, i.e., by more than 76% for a stirring time of 6 h and more than 66% for a stirring time of 8 h;
- Cd extraction is reduced in both chitosan solutions and is not significantly influenced by either increasing chitosan solution concentration or increasing stirring time;
- In Cr, increasing the concentration of the chitosan solution improves the extraction by more than 50% at a stirring time of 2 h but does not change significantly when increasing the stirring time to 4 h, 6 h, or 8 h.

From these investigations, it can be concluded that the extraction of metals is not significantly influenced by increasing the concentration of chitosan solution. This was also demonstrated by Hidary [41], who found that the extraction of Cd (60%) survived regardless of the dose of chitosan used (2%, 4%, and 6%). However, some research has shown that the removal of copper (30.99–43.49%) and nickel (56.22–63.34%) increased with increasing chitosan concentration from 0.1 to 0.4 g/L, but the increase was not proportional, and when the chitosan dose was sufficient for the soil requirement, the influence was very small. The critical extraction level was 0.3 g/L [105].

#### 3.4.3. Extraction of Heavy Metals from Humus Solution

In the case of using a humus solution of 1% concentration as an extraction medium (Figure 5), it can be said that this time, the extraction of Cu, Zn, and also Cr is favoured approximately equally. Comparing the experimental results obtained for extraction in a 1% humus solution vs. extraction in water, it can be said that there is a relatively small decrease for Cu and Cr but a significant decrease for Zn. Thus, the amount of Zn extracted using 1% humus solution is 8–10 times lower than that extracted in water, with the values also varying according to the stirring time.

Increasing the stirring time has a negative effect on Zn extraction, with a reduction of 35% and more than 65%, respectively, compared to the amount extracted under stirring conditions for 2 h. A similar trend is observed for Cr. In this case, increasing the stirring time leads to a reduction of Cr by 22–25% compared to the amount extracted under 2 h stirring conditions.

In the case of using 3% humus solution as an extraction medium (Figure 5), Cr extraction can be said to be slightly improved as a result of increasing the humus solution concentration, but Cu and especially Zn extraction are significantly reduced compared to the variant using a 1% humus solution.

Cd extraction in 1% and 3% humus solutions is reduced, respectively, with experimental values close to those recorded when using water or chitosan solutions. Neither the concentration of the solution nor the stirring time have a significant influence on the Cd extraction process.

In terms of Pb, its extraction in humus solutions is null.

Increasing the stirring time when using a humus solution of 3% concentration does not indicate a linear influence of this parameter on the extraction process, with both Cu and Cr showing maximum values under stirring conditions for 6 h, while for Pb, Zn, and Cd the evolution is random.

#### 3.4.4. Extraction of Heavy Metals from Malic Acid Solution

Using malic acid solutions of 1% and 3% concentration, several improvements to the extraction process were observed (Figure 6):

- Cu extraction increases by up to 10 times compared to using water, chitosan solution, or humus solution if 1% malic acid solution is used, and by more than 20 times if 3% malic acid solution is used. Increasing the concentration of the malic acid solution from 1% to 3% improves the Cu extraction process by 75–165%; this improvement is also influenced by the stirring time. It is noted, however, that in the 1% malic acid solution, as the stirring time increases, there is a continuous reduction in the amount of Cu by 10–24%, compared to the amount recorded with a stirring time of 2 h. In contrast to this downward trend for the 1% malic acid solution, when using the 3% malic acid solution, increasing the stirring time results in a uniform-continuous increase in the amount of Cu extracted by 10–15% compared to the amount extracted with a 2 h stirring time;
- Pb extraction is also dramatically improved, more so as the concentration of the malic acid solution increases from 1% to 3%. Thus, while in the case of water, chitosan, or humus solutions, the quantities of Pb extracted were in the range of unit or subunit values (maximum 1.55 mg L^−1^ or even 0 for humus solutions), under these conditions, values in the range 3–7.4 mg L^−1^ are recorded for the 1% malic acid solution and in the range 25.27–34.98 mg L^−1^ for the 3% malic acid solution. Analysing the influence of stirring time on Pb extraction, two different tendencies are observed, similar to the Cu case, depending on the solution concentration. In the 1% solution, the increase in stirring time has a negative effect, with the amounts of Pb decreasing continuously from 7.4 mgL^−1^ (stirring time of 2 h) to 3.02 mg L^−1^ (stirring time of 8 h), while in the 3% malic acid solution, the increase in stirring time causes a continuous increase of 15–38% compared to the amount extracted with a stirring time of 2 h;
- Zn extraction in malic acid solutions is lower compared to extraction in water, chitosan solution, or humus solution. However, it can be appreciated that the use of a more concentrated malic acid solution (3%) is beneficial in terms of Zn extraction possibilities;
- Cr extraction in malic acid solutions is not significantly different from the cases using water, chitosan, or humus solutions, with values in the range of 1.11–3.97 mg L^−1^. For this element, a negative effect of increasing the concentration of the malic acid solution and increasing the stirring time can be appreciated;
- In the case of Cd, an improvement in the process is appreciated with increasing concentration of malic acid solution, but the recorded values (mg L^−1^) remain subunit. It should be noted, however, that in the case of the use of 3% malic acid solution, the values recorded exceed 0.5 mg L^−1^, falling within the range 0.67–0.78 mg L^−1^, values which were not recorded in the previous cases of the use of other extraction solutions, the maximum value being 0.32 mg L^−1^ in the case of extraction in water with a stirring time of 6 h. Even for the case of using 1% malic acid solution, slightly improved values are recorded, i.e., 0.49 mg L^−1^ for 2 h stirring time. A similarity with the previous situations can be appreciated in the tendency for reduction of Cd extraction with increasing stirring time in a 1% malic acid solution, as opposed to the positive influence of increasing Cd quantity with increasing stirring time in a 3% solution.

Even though increasing the concentration of malic acid solution from 1% to 3% improves the extraction process, extraction was performed at a maximum concentration of 3% because studies by Zhao [1] demonstrated a successful lead removal rate of 70% at a 3% concentration of malic acid solution, and an increase in the concentration of washing solution (4–6%) showed stability even though the concentration increased to 6%.

#### 3.4.5. Extraction of Heavy Metals from Gluconic Acid Solution

Using gluconic acid solutions of 1% and 3% concentration, respectively, several aspects are observed (Figure 7):

- The amount of Cu and Zn extracted represents the highest values compared to the other metals analysed, and increasing the concentration of the gluconic acid solution increases the amount of Zn extracted, even by 50%;
- A positive influence is the increase in stirring time in both 1% and 3% gluconic acid solutions. Overall, by analysing the values obtained experimentally after the 3% malic acid solution under conditions of 4–8 h stirring time, gluconic acid solutions are, of all the solutions analysed, the most favourable medium for Zn extraction. So far, the values recorded were 18.03 mg L^−1^ (malic acid solution 3%, 4 h stirring time) and 17.75 mg L^−1^, respectively, for the situations using malic acid solution 3%, 6 h, and 8 h stirring time, respectively, in the other solutions (water). In the case of a 3% gluconic acid solution, the values recorded reach and exceed 14.98 mg L^−1^, increasing with increasing stirring time;
- Except for favourable cases of Pb extraction in malic acid solution and in 3% gluconic acid solution, the values recorded show better extraction compared to the results obtained for water, chitosan, or humus solution, for which subunit values were generally recorded, with a maximum of 1.55 mg L^−1^ (3% chitosan solution, 4 h stirring time). In a 3% gluconic acid solution, values of 6.61–9.30 mg L^−1^ are recorded, with a tendency to increase with increasing stirring time;
- A similar behaviour is recorded for Cu: quantitatively, the recorded values, although lower compared to malic acid solution, are higher than for water, chitosan solution, or humus solution. The extraction process is favoured by a higher concentration of gluconic acid solution (3%) resulting in an amount of Cu extracted 2–5 times higher compared to the amount of metal extracted when using a 1% concentration solution. However, it is not possible to assess the continuously favourable or unfavourable influence of increasing the stirring time on the amount of Cu extracted from the soil;
- Quantitatively, Cr and Cd keep approximately similar range limits to those recorded for all the other solutions analysed, the values recorded being subunitary, with a maximum of 0.48 mg L^−1^ (gluconic acid solution 3%, 6 h stirring time) in the case of Cd and unitary in the case of Cr, with a maximum of 3.58 mg L^−1^ (gluconic acid solution 3%, 4 h stirring time).

The results showed that some metals were better extracted in a higher concentration of 3% washing solution (Pb, Cu, and Zn), and the extractability of Cd and Cr was not influenced by the concentration of gluconic acid. Studies by Fischer [72] reported that the extractability of Pb was almost unaffected by the concentration of gluconic acid. In general, the 10% solution was less efficient at mobilising the metal than the 5% solution. Pb, Zn, Cr, and Cu were mobilised by the 5% D-gluconic acid solution. In the case of Ni and Cd, the 2% solution was slightly more efficient, and the 10% solution was efficient for Cd [72].

However, it should be noted that the soil analysed contains different amounts of Pb, Cu, Zn, Cd, and Cr. Therefore, for a more conclusive analysis of the metal extraction possibilities, using the different extraction solutions and varying the stirring time, the indicator that allows a unitary comparison of the processes is its yield.

### 3.5. Extraction Process Efficiency

**Figure 8:**
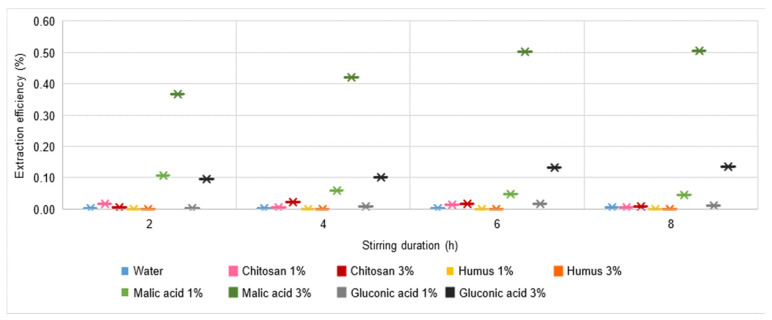
Pb extraction yield in water, chitosan solution, humus solution, malic acid solution, and gluconic acid solution.

Analysing the yield of the Pb extraction process (Figure 8), it is extremely low, regardless of the extraction solution used or the stirring time. More precisely, all the values of this indicator are subunits, with the highest values of 0.5% being recorded for the use of a 3% malic acid solution with stirring times of 6 h and 8 h, respectively.

All these observations lead to the conclusion that none of the solutions used are suitable for Pb extraction. A low efficiency (4%) has been reported in the case of using humus solution [34], and in the case of gluconic acid, higher efficiencies have been reported: 30% [65] and 80% [72], but at pH = 12–13. This may be because Pb ions exist as stable compounds in the soil, and the substances used are not capable of destroying the structure and do not have sufficient complexing capacity to remove Pb ions from the soil under investigation.

**Figure 9:**
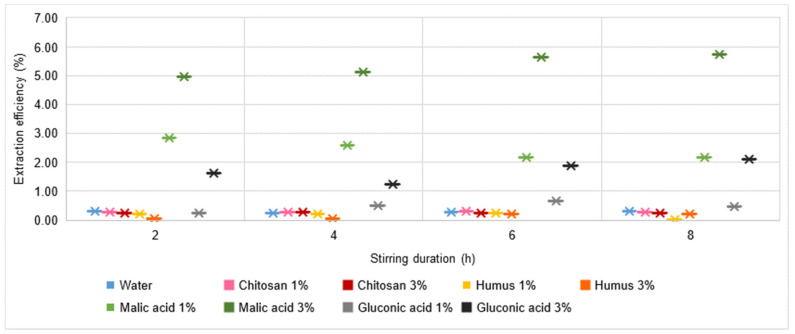
Cu extraction yield in water, chitosan solution, humus solution, malic acid solution, and gluconic acid solution.

**Figure 10:**
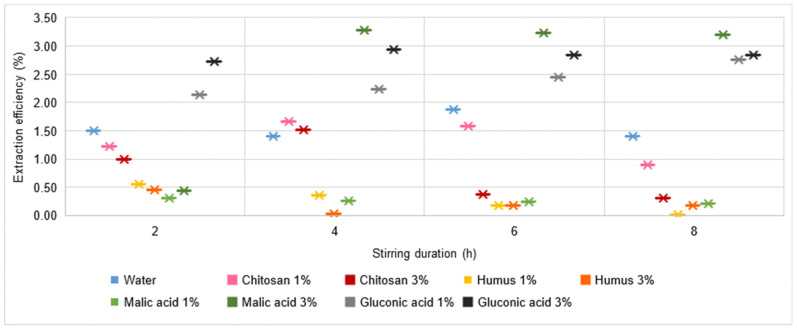
Zn extraction yield in water, chitosan solution, humus solution, malic acid solution, and gluconic acid solution.

A similar, slightly improved behaviour is found for Cu and Zn (Figure 9 and Figure 10). The Cu extraction efficiency reaches the highest values of more than 5% for the use of a 3% malic acid solution with a relatively long stirring time (min. 4 h). Values higher than 1% are reached only when using 1% malic acid or 3% gluconic acid solutions. All in all, the solutions analysed cannot be judged to be favourable for Cu extraction from soil.

For the case of Zn, although extraction yield values higher than 1% are recorded for several solutions used (water, 1% or 3% chitosan solution, 3% malic acid solution, and 1% or 3% gluconic acid solution), the maximum extraction yield is only 3.28% for the situation using 3% malic acid solution with a stirring time of 4 h. Overall, the solutions analysed cannot be considered favourable for the extraction process from the soil in the case of Zn.

**Figure 11:**
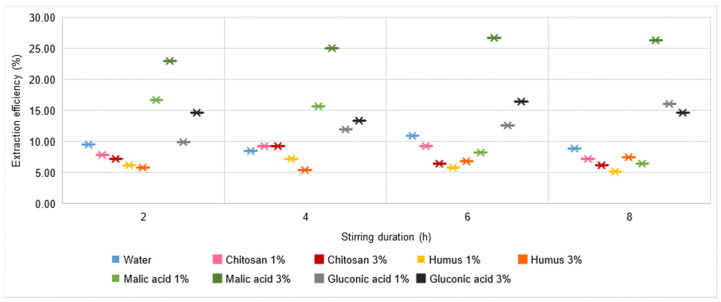
Cd extraction yield in water, chitosan solution, humus solution, malic acid solution, and gluconic acid solution.

In the case of Cd (Figure 11), a significant increase in extraction yield is observed. Thus, all process yield values are above 5%. The best results, in the range 22.95–26.71%, are recorded for the use of 3% malic acid solution, for which it can also be said that an increase in the stirring time has beneficial effects. However, these beneficial effects seem to have a limited evolution at a maximum threshold, and it is possible that an increase in stirring time does not contribute effectively to achieving a cost-benefit balance. Based on the experimental data, it can be judged that, from the point of view of Cd extraction yield from soil, the most favourable of all the variants analysed would be the use of a 3% malic acid solution and a stirring time in the range of 6–8 h.

**Figure 12:**
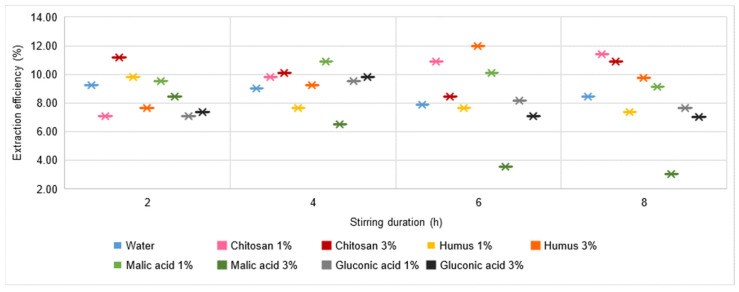
Cr extraction yield in water, chitosan solution, humus solution, malic acid solution, and gluconic acid solution.

Regarding the Cr extraction process (Figure 12), in all cases analysed, there was a yield of at least 3%. The calculated process yield values indicate a much closer placement to each other than for the process yields for the other metals. However, it is not possible to identify an optimal solution used to achieve maximum yield, with values higher than 10% being recorded for the use of 3% chitosan solution with a stirring time of either 2 h or 8 h, 1% chitosan solution with a stirring time of 6 h or 8 h, punctually for the use of 1% malic acid solution and a stirring time of 4 h, and 3% humus solution with a stirring time of 6 h. Of all these cases considered favourable (i.e., with a yield of min. 10%), the only one for which a trend also influenced by stirring time is identified is the case of the 1% chitosan solution, for which it can be said with certainty that an increase in stirring time leads to an increase in extraction process yield. At the same time, analysing the evolution of the process yield in the case of the use of 3% humus solution, the values increase with increasing stirring time, showing a maximum for a stirring time of 6 h, after which the trend is decreasing. This shows that the stirring time influences the process, and it is necessary to identify the optimal stirring time.

The extraction efficiency of zinc (1.5%) using water as an extraction agent is much lower than that obtained by other researchers in the literature (6.7%), while cadmium (11%) shows considerably higher values than the results obtained (1.55%) [106,107].

The washing efficiency by humus extraction solution of Pb (0%), Cu (0.02–0.23%), and Cd (5.1–7.5%) is considerably lower than the results obtained by other researchers for the investigated metals: Cd: 20–40% at pH = 3 [108]; 25% [35]; 35–75% [54]; 36–69% at pH = 5 [108]; Cu: 20–40% at pH = 3 [108]; 5–28% at pH = 5 [106]; 38% [34].

Liu et al. [2] obtained similar results regarding the extraction efficiency of Pb (0%) in the malic acid extraction solution, while other studies report 19% [66]. One explanation is that there are different conditions: the initial concentration of the contaminant in the soil; the solid:liquid ratio used; and the concentration of the extraction solution.

In the chitosan extraction solution, a much lower efficiency of Zn (1–1.15%) or Cd (6.1–9.2%) was found than in other researchers’ studies: Zn: 16.9%; 63%; and Cd: 24%; 92%; 93%; 100% [41,80,104,109,110].

All the metals investigated show much lower extraction yields when using the gluconic acid wash solution (Pb: 0%; Cd: 9.9–16.4%) than those presented in the literature: Pb: 30%; Cd: 34 [65]; or Pb: 80%; Cd: 63% [72]. Some researchers suggest that washing efficiency depends not only on pH but also on the stirring time [108,111]. Efficiency increases with increasing pH, with the optimum value being five [81,83], but above this value, efficiency decreases [108]. Extraction of heavy metals from soil has been performed by some researchers using an extraction time of 24 h [1,2], 48 h [67], or even 58 h [19] in research, but there are studies conducted on 2 h of extraction, achieving high extraction yields (e.g., Cu: 30.5%) [112]. In most cases, a high and ascending extraction is observed in the first hours of the experiments, followed by a linear extraction [19]. In the experiments performed, the maximum extraction time was 8 h for two reasons: (i) the results at 8 h are lower or similar to those obtained at 6 h, and a longer stirring interval is not justified; and (ii) in order to perform a comparative analysis with other research in the field.

## 4. Conclusions

The results showed that the soil analysed is contaminated with heavy metals, being characterised as luteous-coarse sand with a strongly acidic pH, without alkaline-earthy carbonates, with a good supply of nutrients but poor in humus.

Analysing the experimental results obtained at a first assessment, it could be said that any of the proposed solutions allows the extraction of the metals Pb, Cu, Zn, Cd, and Cr from the soil to a greater or lesser extent, depending on the type of metal and the stirring time. Thus, for the use of water, it would be said that the most favourable results are for Zn extraction; for the use of humus and chitosan solutions, Cu, Cr, and Zn extraction would be favorable; malic acid solutions would be suitable for Cu and Pb extraction; and gluconic acid solutions for Cu and Zn.

However, this first impression turns out to be inconclusive. Since the concentrations of Pb, Cu, Zn, Cd, and Cr in the analysed soil vary within wide limits, from 11.68 mg kg^−1^ soil Cd to 27,660 mg kg^−1^ soil Pb, and therefore the availability of extraction is very variable, an analysis of the yield of the extraction processes is more conclusive.

Thus, looking at the yield of the Pb extraction process, it can be said that none of the proposed solutions are suitable, with the maximum value of the indicator being 0.5%. The results for Cu and Zn are not spectacular either, with the yield of the extraction process of these metals being a maximum of 5.72% for Cu and 3.28% for Zn. Slightly improved values are recorded for Cr, for which the maximum yield is 11.97% (humus solution 3%, 6 h stirring time). Cd is an exception to the above, in which case yield values even above 25% are recorded if a 3% malic acid solution is used. Therefore, it is considered that, from case to case, the type of extraction solution as well as the nature of the metal influence the mechanism of the depollution process, i.e., the ability of the fine soil granules to free themselves from the pollutant metal that has adhered to them.

Even if the extraction efficiency is relatively low, the research does not represent a new direction of approach, with the mention that probably the modification of the concentration of the solutions/modification of the extirpation method could lead to a better efficiency of the solute depollution, all this in more environmentally friendly conditions and at the lowest possible costs. Therefore, in the future, it will be necessary to carry out some research in terms of changing the concentration of washing solutions and identifying new washing agents in order to increase the efficiency of the extraction of heavy metals by washing.

In view of the above, it is considered that more studies are needed in the future in order to develop new methods that can effectively lead to the removal of heavy metals from contaminated soil, and a comparative analysis of the techniques used for investigations (e.g., XRD, FTIR, and ICP) should be carried out in the future to identify a direction for further research.

## Figures and Tables

**Table 1:** Values for permitted concentrations according to Order No. 756 [mg kg^−1^] [13].

Heavy Metals	Normal Value	Soil Type			
		Sensitive Soils		Less Sensitive Soils	
		Alert Threshold	Intervention Threshold	Alert Threshold	Intervention Threshold
**Pb**	20	50	100	250	1000
**Cu**	20	100	200	250	500
**Zn**	100	300	600	700	1500
**Cd**	1	3	5	5	10
**Cr**	30	100	300	300	600

**Table 2:** Sample coding.

Extraction Solution	Extraction Solution Concentration	Solid/Liquid Ratio (S/L)	Stirring Time [h]			
			2	4	6	8
Water (A)	-	1:4	A, 1:4, 2	A, 1:4, 4	A, 1:4, 6	A, 1:4, 8
Chitosan (C)	1%	1:4	C, 1%, 1:4, 2	C, 1%, 1:4, 4	C, 1%, 1:4, 6	C, 1%, 1:4, 8
	3%	1:4	C, 3%, 1:4, 2	C, 3%, 1:4, 4	C, 3%, 1:4, 6	C, 3%, 1:4, 8
Humus (H)	1%	1:4	H, 1%, 1:4, 2	H, 1%, 1:4, 4	H, 1%, 1:4, 6	H, 1%, 1:4, 8
	3%	1:4	H, 3%, 1:4, 2	H, 3%, 1:4, 4	H, 3%, 1:4, 6	H, 3%, 1:4, 8
Malic acid (AM)	1%	1:4	AM, 1%, 1:4, 2	AM, 1%, 1:4, 4	AM, 1%, 1:4, 6	AM, 1%, 1:4, 8
	3%	1:4	AM, 3%, 1:4, 2	AM, 3%, 1:4, 4	AM, 3%, 1:4, 6	AM, 3%, 1:4, 8
Gluconic acid (AG)	1%	1:4	AG, 1%, 1:4, 2	AG, 1%, 1:4, 4	AG, 1%, 1:4, 6	AG, 1%, 1:4, 8
	3%	1:4	AG, 3%, 1:4, 2	AG, 3%, 1:4, 4	AG, 3%, 1:4, 6	AG, 3%, 1:4, 8

**Table 3:** Selected physicochemical soil properties.

Soil Properties		Value
pH		4.67
Texture		coarse loamy-sand (UG)
Hygroscopicity coefficient (CH)		4.8 [% g/g]
Content of hydrostable macrostructural elements (HA)		9 [% g/g]
Content of hydrostable microstructural elements (D)		17 [% g/g]
Structural Instability Index (SI)		1.95 [-]
C_org_		1.23 [%]
Humus		2.12 [%]
Micronutrients	N_t_	0.107 [%]
	P_AL_	6 [mg kg^−1^]
	K_AL_	26 [mg kg^−1^]

**Table 4:** Cation exchange property values obtained.

Ca^2+^	Mg^2+^	K^+^	Na^+^	SB	H^+^	T (SB + SH)	Ca^2+^	Mg^2+^	K^+^	Na^+^	H^+^	V	Total Soluble Salt Content
me/100 g soil							wt% from T						mg/100 g soil
1.29	0.20	0.13	0.01	1.63	9.07	10.70	12.1	1.8	1.2	0.1	84.8	15.2	10.70

**Table 5:** Heavy metal concentration determined on soil samples and indication of exceeding the values set by Order 756/1997 [13].

Heavy Metals	Pb	Cu	Zn	Cd	Cr
Measured value [mg kg^−1^]	27,660	5590	2199	11.68	146
How many times does the measured value exceed the normal value	1383	279.5	21.99	11.68	5.33
How many times does the measured value exceed the alert threshold for less sensitive soils	110.6	22.36	3.14	2.33	*
How many times does the measured value exceed the intervention threshold for less sensitive soils	27.66	11.18	1.46	1.16	*

* The measured value is below the alert/intervention threshold value but exceeds the normal value.

**Table 6:** pH of the extraction solutions depending on the substance and concentration used.

Extraction Solution	Water	Chitosan 1%	Chitosan 3%	Humus 1%	Humus 3%	Malic Acid 1%	Malic Acid 3%	Gluconic Acid 1%	Gluconic Acid 3%
pH	6.8 ± 0.1	4.9 ± 0.1	5.2 ± 0.1	5.3 ± 0.1	5.8 ± 0.1	3.8 ± 0.1	3.4 ± 0.1	4.8 ± 0.1	4.5 ± 0.1

## Data Availability

Not applicable.

## References

[1] Zhao M., Yan H., Liu C., Li C., Li S., Gao H. (2022). Heavy metal lead (Pb) removal in contaminated soils using citric acid and malic acid as washing agents. Res. Sq..

[2] Liu H., Chen P., Wang H., Yang Y., Wu Y. (2023). Remediation of Cu-, Zn-, and Pb-Contaminated Soil Using Different Soil Washing Agents: Removal Efficiencies and Mechanisms. Water Air Soil Pollut..

[3] Liu C., Zhang H.X. (2022). Modified-biochar adsorbents (MBAs) for heavy-metal ions adsorption: A critical review. J. Environ. Chem. Eng..

[4] Wu B., Wang Z., Zhao Y., Gu Y., Wang Y., Yu J., Xu H. (2019). The performance of biochar-microbe multiple biochemical material on bioremediation and soil micro-ecology in the cadmium aged soil. Sci. Total Environ..

[5] Ma H., Li X., Wei M., Zeng G., Hou S., Li D., Xu H. (2020). Elucidation of the mechanisms into effects of organic acids on soil fertility, cadmium speciation and ecotoxicity in contaminated soil. Chemosphere.

[6] Piatak N.M., Parsons M.B., Seal R.R. (2015). Characteristics and environmental aspects of slag: A review. J. Appl. Geochem..

[7] Wang J.P., Erdenebold U. (2020). A study on reduction of copper smelting slag by carbon for recycling into metal values and cement raw material. Sustainability.

[8] Moon D.H., Lee J.-R., Wazne M., Park J.-H. (2012). Assessment of soil washing for Zn contaminated soils using various washing solutions. J. Ind. Eng. Chem..

[9] Lajayer B.A., Ghorbanpour M., Nikabadi S. (2017). Heavy metals in a contaminated environmenta: Destiny of secondary metabolite biosynthesis, oxidative status and phytoextraction in medicinal plants. Ecotoxicol. Environ. Saf..

[10] Khalid S., Shahid M., Niazi N.K., Murtaza B., Bibi I., Dumat C. (2017). A comparison of technologies for remediation of heavy metal contaminated soils. J. Geochem. Explor..

[11] Gorospe J. (2012). Growing Greens and Soiled Soil.

[12] EEA Progress in Management of Contaminated Sites. https://www.eea.europa.eu/data-and-maps/indicators/progress-in-management-of-contaminated-sites-3/assessment.

[13] (1997). Order No. 756 of 3 November 1997 for the Approval of the Regulation on Environmental Pollution, Assesment. Official Gazzate No. 303 Bis of 6 November 1997.

[14] Tangahu B.V., Abdullah S.R.S., Basri H., Idris M., Anuar N., Mukhlisin M. (2011). A Review on Heavy Metals (As, Pb, and Hg) Uptake by Plants through Phytoremediation. Int. J. Chem. Eng..

[15] NEPA (2020). Annual Report on the State of the Environment in Romania in 2020.

[16] Elekes C.C., Hernandez Soriano M.C. (2014). Assessment of Historical Heavy Metal Pollution of Land in the Proximity of Industrial Area of Targoviste, Romania, Environmental Risk Assessment of Soil Contamination.

[17] Vrînceanu N.O., Motelică D.M., Dumitru M., Toti M., Gamenţ E., Tănase V. Aspects Concerning soil Pollution with Heavy Metals in Copşa Mică Area. Proceedings of the International Conference “Soil under Global Change”.

[18] Lăcătuşu R., Cârstea S., Lungu M., Kovacsovics B., Lazăr R. (2002). Soil pollution with cyanides and heavy metals in the Baia Mare area; ecological reconstruction. Soil Sci..

[19] Sun Y., Zhang P., Li Z., Chen J., Ke Y., Hu J., Liu B., Yang J., Liang S., Su X. (2022). Iron-calcium reinforced solidification of arsenic alkali residue in geopolymer composite: Wide pH stabilization and its mechanism. Chemosphere.

[20] Scanferla P., Marcomini A., Pellay R., Girotto P., Zavan D., Fabris M., Collina A. (2012). Remediation of a Heavy Metals Contaminated Site with a Botanical Garden: Monitoring Results of the Application of an Advanced S/S Technique. Chem. Eng. Trans..

[21] Sun L., Wu Q., Liao K., Yu P., Cui Q., Rui Q., Wang D. (2016). Contribution of heavy metals to toxicity of coal combustion related fine particulate matter (PM2.5) in *Caenorhabditis elegans* with wild-type or susceptible genetic background. Chemosphere.

[22] Sinha S., Mishra R.K., Sinam G., Mallick S., Gupta A.K. (2013). Comparative Evaluation of Metal Phytoremediation Potential of Trees, Grasses, and Flowering Plants from Tannery-Wastewater-Contaminated Soil in Relation with Physicochemical Properties. Soil Sediment Contam. Int. J..

[23] Bahemmat M., Farahbakhsh M., Kianirad M. (2016). Humic substances-enhanced electroremediation of heavy metals contaminated soil. J. Hazard. Mater..

[24] Golia E.E., Angelaki A., Giannoulis K.D., Skoufogianni E., Bartzialis D., Cavalaris C., Vleiora S. (2021). Evaluation of soil properties, irrigation and solid waste application levels on Cu and Zn uptake by industrial hemp. Agron. Res..

[25] Soleimani M., Hajabbasi M.A., Afyuni M., Akbar S. (2010). Comparison of Natural Humic Substances and Synthetic Ethylenediaminetetraacetic Acid and Nitrilotriacetic Acid as Washing Agents of a Heavy Metal–Polluted Soil. J. Environ. Qual..

[26] Lestan D., Luo C., Li X. (2008). The use of chelating agents in the remediation of metal-contaminated soils: A review. Environ. Pollut..

[27] Marchiol L., Assolari S., Sacco P., Zerbi G. (2004). Phytoextraction of heavy metals by canola (*Brassica napus*) and radish (*Raphanus sativus*) grown on multicontaminated soil. Environ. Pollut..

[28] Ali H., Khan E., Sajad M.A. (2013). Phytoremediation of heavy metals-concepts and Applications. Chemosphere.

[29] Sidhu G.P.S. (2016). Heavy metal toxicity in soils: Sources, remediation technologies and challenges. Adv. Plants Agric. Res..

[30] Gong Y., Zhao D., Wang Q. (2018). An overview of field-scale studies on remediation of soil contaminated with heavy metals and metalloids: Technical progress over the last decade. Water Res..

[31] Chen X., Achal V. (2019). Biostimulation of carbonate precipitation process in soil for copper immobilization. J. Hazard. Mater..

[32] Gusiatin Z.M. (2018). Novel and Eco-Friendly Washing Agents to Remove Heavy Metals from Soil by Soil Washing. Environ. Anal. Ecol. Stud..

[33] Kim M.-S., Koo N., Kim J.-G., Lee S.-H. (2021). Effects of Washing Solution, Washing Time, and Solid-Solution Rate on the Maximum Heavy Metals Removal Efficiency. Appl. Sci..

[34] Borggaard O.K., Hansen H.C.B., Holm P.E., Jensen J.K., Rasmussen S.B., Sabiene N., Steponkaite L., Strobel B.W. (2009). Experimental assessment of using soluble humic substances for remediation of heavy metal polluted soils. Soil Sediment Contam..

[35] Borggaard O.K., Holm P.E., Jensen J.K., Soleimani M., Strobel B.W. (2011). Cleaning heavy metal contaminated soil with soluble humic substances instead of synthetic polycarboxylic acids. Acta Agric. Scand. Sect. B—Soil Plant Sci..

[36] Borggaard O.K., Holm P.E., Strobel B.W. (2019). Potential of dissolved organic matter (DOM) to extract As, Cd, Co, Cr, Cu, Ni, Pb and Zn from polluted soils: A review. Geoderma.

[37] Yang T., Hodson M.E. (2019). Investigating the use of synthetic humic-like acid as a soil washing treatment for metal contaminated soil. Sci. Total Environ..

[38] Cheng S., Lin Q., Wang Y., Luo H., Huang Z., Fu H., Chen H., Xiao R. (2020). The removal of Cu, Ni, and Zn in industrial soil by washing with EDTA-organic acids. Arab. J. Chem..

[39] Race M., Marotta R., Fabbricino M., Pirozzi F., Andreozzi R., Cortese L., Giudicianni P. (2016). Copper and zinc removal from contaminated soils through soil washing process using ethylenediaminedisuccinic acid as a chelating agent: A modeling investigation. J. Environ. Chem. Eng..

[40] Mohanty B., Mahindrakar A.B. (2011). Removal of Heavy Metal by Screening Followed by Soil Washing from Contaminated Soil. IJTES.

[41] Hydari S., Sharififard H., Nabavinia M., Reza Parvizi M. (2012). A comparative investigation on removal performances of commercial activated carbon, chitosan biosorbent and chitosan/activated carbon composite for cadmium. Chem. Eng. J..

[42] Yi N., Wu Y., Fan L., Hu S. (2019). Remediating Cd-Contaminated Soils Using Natural and Chitosan-Introduced Zeolite, Bentonite, and Activated Carbon. Pol. J. Environ. Stud..

[43] Desbrieres J., Guibal E. (2011). Sorption Processes and Pollution: Conventional and Non-Conventional Sorbents for Pollutant Removal from Wastemasters.

[44] Mahaweero T. (2013). Extraction of Heavy Metals from Aqueous Solutions Using Chitosan/Montmorillonite Hybrid Hydrogels. Master’s Thesis.

[45] Sewvandi G.A., Adikary S.U. (2011). Removal of Heavy Metals from Wastewater Using Chitosan. Soc. Soc. Manag. Syst. Internet J..

[46] Perminova I.V., Hatfield K. (2005). Use of Humic Substances to Remediate Polluted Environments: From Theory to Practice.

[47] Boguta P., Sokolowska Z. (2013). Interactions of humic acids with metals. Acta Agrophys..

[48] Rahman M.A., Hasan M.A., Rahim A., Alam A.M.S. (2010). Characterization of Humic Acid from the River Bottom Sediments of Burigonga: Complexation Studies of Metals with Humic Acid. Pak. J. Anal. Environ. Chem..

[49] Pehlivan E., Arslan G. (2006). Uptake of Metal Ions on Humic Acids. Energy Sources A Recovery Util. Environ. Eff..

[50] Alberts J.J., Filip Z. (1998). Metal binding in estuarine humic and fulvic acids: FTIR analysis of humic acids—Metal complexes. Environ. Technol..

[51] Tang H., Shuai W., Wang X., Liu Y. (2017). Extraction of rare earth elements from a contaminated cropland soil using nitric acid, citric acid, and EDTA. Environ. Technol..

[52] Jelusic M., Lestan D. (2014). Effect of EDTA washing of metal polluted garden soils. Part I: Toxicity hazards and impact on soil properties. Sci. Total Environ..

[53] Kim E.J., Jeon E.K., Baek K. (2016). Role of reducing agent in extraction of arsenic and heavy metals from soils by use of EDTA. Chemosphere.

[54] Gusiatin Z.M., Kulikowska D., Klik B. (2020). New-generation washing agents in remediation of metal-polluted soils and methods for washing effluent treatment: A review. Int. J. Environ. Res. Public Health.

[55] Cao Y., Zhang S., Wang G., Li T., Xu X., Deng O., Zhang Y., Pu Y. (2017). Enhancing the soil heavy metals removal efficiency by adding HPMA and PBTCA along with plant washing agents. J. Hazard. Mater..

[56] Zheng X.-J., Li Q., Peng H., Zhang J.-X., Chen W.-J., Zhou B.-C., Chen M. (2022). Remediation of Heavy Metal-Contaminated Soils with SoilWashing: A Review. Sustainability.

[57] Guo X., Zhao G., Zhang G., He Q., Wei Z., Zheng W., Qian T., Wu Q. (2018). Effect of mixed chelators of EDTA, GLDA, and citric acid on bioavailability of residual heavy metals in soils and soil properties. Chemosphere.

[58] Zhang H., Xu Y., Kanyerere T., Wang Y.-S., Sun M. (2022). Washing Reagents for Remediating Heavy-Metal-Contaminated Soil: A Review. Front. Earth Sci..

[59] Ubner M. (2004). Interaction of Humic Substances with Metal Cations.

[60] Gusiatin Z.M., Klimiuk E. (2012). Metal (Cu, Cd and Zn) removal and stabilization during multiple soil washing by saponin. Chemosphere.

[61] Huang G.Y., You J.W., Zhou X.P., Ren C., Islam M.S., Hu H.Q. (2020). Effects of low molecular weight organic acids on Cu accumulation by castor bean and soil enzyme activities. Ecotoxicol. Environ. Saf..

[62] Gomez-Garrido M., Navarro J.M., Navarro F.J.M., Cano A.F. (2018). The chelating effect of citric acid, oxalic acid, amino acids and Pseudomonas fluorescens bacteria on phytoremediation of Cu, Zn, and Cr from soil using *Suaeda vera*. Int. J. Phytoremediat..

[63] Qiao D.M., Lu H.F., Zhang X.X. (2020). Change in phytoextraction of Cd by rapeseed (*Brassica napus* L.) with application rate of organic acids and the impact of Cd migration from bulk soil to the rhizosphere. Environ. Pollut..

[64] Sun Y., Luo T., Zhong S., Zhou F., Zhang Y., Ma Y., Fu Q. (2021). Long-term effects of low-molecular-weight organic acids on remobilization of Cd, Cr, Pb, and As in alkaline coastal wetland soil. Environ. Pollut..

[65] Han R., Dai H., Skuza L., Wei S. (2021). Comparative study on different organic acids for promoting *Solanum nigrum* L. hyperaccumulation of Cd and Pb from the contaminated soil. Chemosphere.

[66] Li Z., Deng H.X., Gong Z.Q., Liu S., Yang Y.T. (2018). Washing efficiency of Cd from contaminated lou soil by saponin and low-molecular-weight organic acids. J. Northwest A F Univ.-Nat. Sci. Ed..

[67] Zhong L., Yang J. (2011). Reduction of Cr(VI) by Malic Acid in Aqueous Fe-Rich Soil Suspensions. Chemosphere.

[68] Ke X., Zhang F.J., Zhou Y. (2020). Removal of Cd, Pb, Zn, Cu in smelter soil by citric acid leaching. Chemosphere.

[69] Jiang H., Li T., Han X., Yang X., He Z. (2012). Effects of pH and low molecular weight organic acids on competitive adsorption and desorption of cadmium and lead in paddy soils. Environ Monit..

[70] Parimal P., Ramesh K., Subhamay B. (2016). Manufacture of gluconic acid: A review towards process intensification for green production. Chem. Eng. Process.

[71] Scheglova N.V., Popova T.V., Druzhinina A.V., Smotrina T.V. (2019). Spectrophotometric study of complexation of cobalt (II) with HEDP in aqueous solutions. J. Mol. Liq..

[72] Fischer K., Bipp H.-P. (2002). Removal of Heavy Metals from Soil Components and Soils by Natural Chelating Agents. Part II. Soil Extraction by Sugar Acids. Water Air Soil Pollut..

[73] Arafat A., Sabrin A.S., Shah M.M., Mohammad M. (2015). Preparation and Characterization of Chitosan from Shrimp shell waste. Int. J. Sci. Eng. Res..

[74] Agarwal R.M., Singh K. (2017). Heavy metal removal from wastewater using various adsorbents: A review. J. Water Reuse Desalin..

[75] Kamari A., Pulford I.D., Hargreaves J.S.J. Chitosan-assisted Phytoextraction of Heavy Metal from Lead/Zinc Tailings Using Lolium Perenne—A Preliminary Study. Proceedings of the 15th International Conference on Heavy Metals in the Environment.

[76] Qasem N.A.A., Mohammed R.H., Lawal D.U. (2021). Removal of heavy metal ions from wastewater: A comprehensive and critical review. Clean Water.

[77] Lu C., Xu Z., Dong B., Zhang Y., Wang M., Zeng Y., Zhang C. (2022). Machine learning for the prediction of heavy metal removal by chitosan-based flocculants. Carbohydr. Polym..

[78] Hou T., Du H., Yang Z., Tian Z., Shen S., Shi Y., Zhang L. (2019). Flocculation of different types of combined contaminants of antibiotics and heavy metals by thermo-responsive flocculants with various architectures. Sep. Purif. Technol..

[79] Herath B.G., Crisler A., Bridges G., Patel D., Pittman S., Todd C.M. (2020). Cadmium and Copper Removal from Aqueous Solutions Using Chitosan-Coated Gasifier Biochar. Front. Environ. Sci..

[80] Wan M.W., Petrisor I.G., Lai H.T., Kim D., Yen T.F. (2004). Copper adsorption through chitosan immobilized on sand to demonstrate the feasibility for in situ soil decontamination. Carbohydr. Polym..

[81] Sur I.M., Micle V., Polyak E.T., Gabor T. (2022). Assessment of Soil Quality Status and the Ecological Risk in the Baia Mare, Romania Area. Sustainability.

[82] Damian G., Damian F., Nasui D., Pop C., Pricop C. (2010). The soils quality from the southern–eastern part of Baia Mare zone affected by metallurgical industry. Carpathian J. Earth Environ. Sci..

[83] Aydinalp C., Marinova S. (2003). Distribution and forms of heavy metals in some agricultural soils. Pol. J. Environ. Stud..

[84] (1984). Soils. Sample Collection for Soil and Agrochemical Studies.

[85] (1998). Soil Quality. Pretreatment of Samples for Psysico-Chemical Analysis.

[86] (1988). Soils. Determination of pH.

[87] (1999). Soil Quality. Determination of pH.

[88] Rusu T., Paulette L., Cacoveanu H., Turcu V. (2007). Physics, Hydrophysics, Environmental Chemistry and Soil Respiration, Research Methods.

[89] Dumitru M., Manea A., Ciobanu C., Dumitru S., Vrînceanu N., Calciu I., Tănase V., Preda M., Rîşnoveanu I., Mocanu V. (2011). Soil Quality Monitoring in Romania.

[90] Technical Agricultural Propaganda Editorial Office (1987). Methodology of Soil Studies. Volumes I–III.

[91] Ştefan D.S., Onose C., Apostol D.G., Bobirică C. (2003). Environmental Quality Control—Practical Laboratory Work.

[92] Bumbu I., Bumbu I., Vîrlan L. (2006). Environmental Control and Monitoring. Laboratory and Practical Course.

[93] (1978). Determination of Hygroscopicity Index.

[94] (1982). Soils. Determination of Organic Carbon and Humus.

[95] de Oliveira E. (2003). Sample preparation for atomic spectroscopy: Evolution and future trends. J. Braz. Chem. Soc..

[96] (1985). Soils. Determination of Nitrogen Content.

[97] (2001). Soil Quality. Determination of Nitrogen Content.

[98] (1982). Soils. Determination of Phosphorus.

[99] (1979). Soils. Determination of Cation Exchange Properties.

[100] Couillard D., Chartier M., Mercier G. (1994). Étude de l’enlèvement du Cd, Cu, Mn et Zn par solubilisation biologique dans les sédiments lacustres fortement contaminés. J. Water Sci..

[101] Côme B., Barres-Lakkemand A., Ricour J., Martin S. (1993). Applications comparatives de méthodes d’ évaluation de risques liés aux sites pollués: Premiers enseignements et perspectives. TSM.

[102] Damian F., Damian G. (2007). Detoxification of Heavy Metal Contaminated Soils. Am. J. Environ. Sci..

[103] Luduşan N. (2007). Efectele Acumulării Metalelor Grele în Soluri Asupra Componentei Biotice Din Depresiunea Zlatna. https://www.uab.ro/reviste_recunoscute/revcad/revcad_2007/27.ludusan_nicolae.pdf.

[104] Damian F., Damian G., Lacătusu R., Macovei G., Iepure G., Năprădean I., Chira R., Kollar L., Rată L., Zaharia C. (2008). Soils from the Baia Mare zone and the heavy metals pollution. Carpathian J. Earth Environ. Sci..

[105] Jiang W., Tao T., Liao Z. (2011). Removal of Heavy Metal from Contaminated Soil with Chelating Agents. J. Soil Sci..

[106] Hu W., Niu Y., Zhu H., Dong K., Wang D., Liu F. (2021). Remediation of zinc-contaminated soils by using the two-step washing with citric acid and water-soluble chitosan. Chemosphere.

[107] Wen J., Xing L., Wang Y. (2019). Chemical and microbiological responses of heavy metal contaminated sediment subject to washing using humic substances. Environ. Sci. Pollut. Res..

[108] Kulikowska D., Gusiatin Z.M., Bułkowska K., Kierklo K. (2015). Humic substances from sewage sludge compost as washing agent effectively remove Cu and Cd from soil. Chemosphere.

[109] Li B., Li M., Zhang P., Pan Y., Huang Z., Xiao H. (2022). Remediation of Cd (II) ions in aqueous and soil phases using novel porous cellulose/chitosan composite spheres loaded with zero-valent iron nanoparticles. React. Funct. Polym..

[110] Xiang J., Lin Q., Yao X., Yin G. (2021). Removal of Cd from aqueous solution by chitosan coated MgO-biochar and its in-situ remediation of Cd-contaminated soil. Environ. Res..

[111] Tripathi N., Choppala G., Singh R.S. (2017). Evaluation of modified chitosan for remediation of zinc contaminated soils. J. Geochem. Explor..

[112] Wang L., Wei J., Yang L., Chen Y., Wang M., Xiao L., Yuan G. (2023). Enhancing Soil Remediation of Cop-per-Contaminated Soil through Washing with a Soluble Humic Substance and Chemical Reductant. Agronomy.

